# Diphtheria Antitoxin in General Practice

**Published:** 1899-03-11

**Authors:** 


### DIPHTHERIA ANTITOXIN IN GENERAL PRACTICE.

A very striking series of cases is published by Dr. Richardson Armstrong,[1] showing the results to be obtained by the use of diphtheria antitoxin in general practice. The list includes over 120 cases of diphtheria occurring among his own patients, but the antitoxin was not administered to all of them, a considerable number of those which appeared to be mild cases not having been injected. What the figures show, then, is that, notwithstanding the fact that the antitoxin was reserved for the more severe cases, those which were injected did better on the average than the apparently mild cases, which were treated by ordinary methods. During the latter half of 1897 42 cases were treated. Of these 22 were severe, and were therefore injected. Two of them died, or a case mortality of 9 per cent., while of the remaining 20 apparently mild cases, which were not injected, four died, a case mortality of 20 per cent. The average fatality for the whole series during this period was 14$\frac{2}{7}$ per cent. During the year 1898 the total number of cases treated was 80. As before, the severe cases, numbering 55, were injected. Only one died, a case mortality of under 2 per cent., while of the remaining 25 apparently mild cases, which were not injected, two died, a case mortality of 8 per cent., the average fatality of the series being 3$\frac{3}{4}$ per cent. Thus we have a very striking demonstration of the utility of antitoxin, the results being better in those which were injected because they were severe than in those which were left alone because they seemed to be mild. The benefit of the treatment is shown also by comparing the results obtained in this general practice, a practice in which a large proportion of the cases (62 per cent. in 1897 and 68$\frac{3}{4}$ per cent. in 1898) were injected, with the results current in this district; for while the average mortality of Dr. Armstrong's cases in the second half of 1897 was 14$\frac{2}{7}$ per cent., and in 1898 was only 3$\frac{3}{4}$ per cent., the report of the medical officer of health for the Rhondda Valley for 1897 shows that the case mortality of those attacked by diphtheria throughout the district averaged 30 per cent. The clinical details given in regard to the progress of the patients in the two groups of cases show how markedly the injections tended to hasten the disappearance of the membrane, and to bring the patients into a state of convalescence. The amount of antitoxin used was 1,500 units of antitoxic serum (Burroughs, Wellcome, and Co.), which was injected under the skin of the abdomen, and the liquid, not the dried, serum was always used. This was generally found to be enough to cure the patient, and in only five out of the 77 cases injected was it found necessary to use a second dose. Dr. Richardson Armstrong is to be congratulated on his results, and the convincing testimony which they afford of the utility of antitoxin in private practice.

[1] Lancet, Mar. 4.

